# Comparative Analysis of Effectiveness of Dolutegravir-Based Regimen (TLD) vs. Efavirenz-Based Regimen (TLE) to Prevent Mother-to-Child Transmission of HIV

**DOI:** 10.7759/cureus.100824

**Published:** 2026-01-05

**Authors:** Monika Kashyap, Aruna Verma, Deepti Pathak, Sandhya Gautam, Mitali Gupta

**Affiliations:** 1 Obstetrics and Gynaecology, Lala Lajpat Rai Memorial Medical College, Meerut, IND; 2 Internal Medicine, Lala Lajpat Rai Memorial Medical College, Meerut, IND; 3 Obstetrics and Gynaecology, CK Birla Hospital, Gurgaon, IND

**Keywords:** art, mtct, nevirapine, pptct, tld regimen, tle regimen

## Abstract

Background: The National AIDS Control Organisation (NACO) in India now recommends a dolutegravir-based regimen (TLD) for pregnant women to prevent mother-to-child transmission (MTCT) of HIV. However, evidence comparing its effectiveness with the previously used Efavirenz-based regimen (TLE) in reducing MTCT remains limited.

Aim: To compare the effectiveness of the TLD and TLE regimens in preventing mother-to-child transmission of HIV.

Methodology: HIV-positive pregnant women initiated on the TLD regimen were categorized as group A, while those receiving the TLE regimen formed group B. All newborns received nevirapine prophylaxis and underwent HIV testing at 6 weeks, 6 months, 12 months, and 18 months of age.

Result: Among the 67 women in group A, no infants tested positive for HIV. In group B, two infants were HIV-positive (MTCT rate 4.6%). The difference in transmission rates showed no statistical significance (χ²= 2.816; p=0.09), but the DTG-based (TLD) regimen showed a trend toward lower MTCT compared to the EFV-based (TLE) regimen. At initiation, the mean CD4 count in the TLD group was 444.5±199.79 cells/mm with a slight decline after 6 months of therapy (CD4 count= 405.09±147.52). The mean birth weight was 2.75±0.39 kg in group A and 2.59±0.46 in group B.

Conclusion: The TLD regimen was associated with a trend towards lower MTCT of HIV compared to the TLE regimen. Thus, continued use of dolutegravir-based therapy is recommended to support national and global goals of eliminating new HIV infections among exposed infants.

## Introduction

According to the WHO 2023 report, an estimated 39.0 million (33.1-45.7 million) people were living with HIV by the end of 2022, including 1.5 million children aged 0-14 years old [1].

In India, the National AIDS Control Organization (NACO) reports a continued decline in adult HIV prevalence (15-49 years) from its peak of 0.55% in 2000 to 0.32% in 2010 and 0.21% in 2021. Despite the relatively low prevalence, the absolute burden remains substantial, with approximately 2.4 million people living with HIV (PLHIV) nationwide [2].

Globally, around 1.2 million women and girls living with HIV become pregnant every year. Without preventive interventions, the risk of mother-to-child transmission (MTCT) during pregnancy, labor, delivery, or breastfeeding ranges from 15% to 45%. Hence, prompt HIV diagnosis must be followed by linkage to lifelong antiretroviral therapy (ART), adherence support to maintain viral suppression, and partner services [3]. This global strategy has contributed to a significant rise in ART uptake over the past decade.

In 2019, the WHO recommended dolutegravir (DTG)-based regimens as the preferred first-line therapy for adults and adolescents [4]. DTG, an integrase strand transfer inhibitor (INSTI), blocks the viral integrase enzyme responsible for inserting HIV DNA into the host genome [5]. It is also effective against HIV-2, which is inherently resistant to efavirenz (EFV). In line with global recommendations, since July 2020, NACO has endorsed the fixed-dose combination of tenofovir disoproxil fumarate 300 mg + lamivudine 300 mg + dolutegravir 50 mg (TLD) as the preferred regimen for HIV-positive pregnant women [6].

Effective prevention of parent-to-child transmission (PPTCT) requires early antenatal HIV testing, timely initiation of ART, institutional delivery, appropriate infant-feeding counselling, infant ART prophylaxis, and access to family planning and reproductive health services. Although DTG is favored for its rapid viral suppression, high genetic barrier to resistance, and low cost [7], limited evidence exists regarding its comparative effectiveness in reducing MTCT when measured against the earlier EFV-based (TLE) regimen.

Therefore, this study was undertaken to compare the effectiveness of TLD versus TLE in preventing MTCT of HIV among exposed infants and to evaluate fetomaternal outcomes associated with TLD therapy during different programmatic phases, providing real-world observational data following the transition in national PMTCT guidelines.

## Materials and methods

Primary objective

To compare the efficacy of TLD and TLE regimens in preventing mother-to-child transmission (MTCT) of HIV.

Secondary objectives

To assess feto-maternal outcomes among HIV-positive pregnant women receiving the TLD regimen and determine the change in CD4 count before initiation of TLD therapy and after six months of treatment.

Study design and study period

This was a retrospective observational before-and-after study that analyzed data from mother-infant pairs enrolled in the PPTCT/PMTCT program at our center.

TLD group (Group A): (n=67) Data were collected for women initiated on TLD between April 2021 and March 2022.

TLE group (Group B): (n=66) Data were retrieved from records of women who received EFV-based ART between April 2015 and March 2017.

Inclusion criteria

Group A (TLD): Included ART-naive pregnant women who were newly started on the TLD regimen and delivered at our center between April 2021 and March 2022.

Group B (TLE): Mother-infant pairs enrolled in PMTCT care from April 2015 onwards who received an EFV-based (TLE) regimen only until discharge.

Exclusion criteria

Women already on TLD or who switched to TLD before the defined period, and cases where infant HIV outcome data were unavailable or undetermined, were excluded.

Methodology

All pregnant women attending antenatal clinics at our center underwent routine booking, detailed history-taking, and complete general, systemic, and obstetric examinations. Standard antenatal investigations, including hemoglobin, VDRL, urine routine/microscopy, OGTT with 75 g glucose, HIV, HBsAg, and HCV, were performed and repeated as required.

Participants were categorized into two groups

Group A (TLD regimen): Women diagnosed as HIV-positive between April 2021 and March 2022 and initiated on the fixed-dose combination of tenofovir disoproxil fumarate 300 mg + lamivudine 300 mg + dolutegravir 50 mg.

Group B (TLE regimen): Data were sourced from a previously conducted study at our center involving women diagnosed or referred as HIV-reactive and managed from April 2015 to March 2017. They received the Tenofovir 300 mg + Lamivudine 300 mg + Efavirenz 600 mg regimen through the ART center and continued the same throughout pregnancy and postpartum. 

For all women, routine antenatal tests, CD4 counts, and HIV viral load testing were performed as per protocol. After delivery, all infants were registered under the PMTCT program and underwent serial HIV screening using PCR and rapid tests at 6 weeks, 6 months, 12 months, and 18 months.

Infant post-exposure prophylaxis

Nevirapine syrup (2 mg/kg) was administered for six weeks if maternal ART had been initiated before 12 weeks of gestation. For 12 weeks, if ART initiation occurred after 12 weeks of gestation. Mothers were counselled about infant feeding options (exclusive breastfeeding vs. replacement feeding) and provided postpartum contraception advice.

Data from both groups were compared for MTCT rates and relevant maternal and neonatal outcomes.

Statistical analysis

Data were entered in Microsoft Excel (MS Office 2010) and analyzed using IBM Corp. Released 2014. IBM SPSS Statistics for Windows, Version 20. Armonk, NY: IBM Corp. Categorical variables were expressed as frequencies and percentages, while continuous variables were summarized as means with standard deviations. Baseline demographic, obstetric, and neonatal characteristics between the two groups were compared using Pearson’s chi-square test.

## Results

A total of 3404 patients were delivered to our institute; of these, the incidence of HIV in delivered patients was 1.9%. The following observations were made.

Demographic parameters of HIV-positive mothers at the time of registration

In study group A, the majority of the mothers (36 out of 67) belonged to the age group of 25-29 years. In terms of parity, out of 67 patients, 31 mothers were nulliparous, 23 mothers were primiparous, 12 were multiparous, and only one was grand multipara (>4).

Most of the patients in group A tested positive for HIV at 24-35 weeks of GA, and only two subjects were detected before 12 weeks of gestation, which is highly in contrast with group B, where 18 subjects were diagnosed before 12 weeks of GA. 

In group A, 55 mothers (82.1%) opted for exclusive breastfeeding, while in group B, only 66.1% opted for breastfeeding. In group A, 13.4%, while in group B, 11.3% of subjects had a CD4 count below 250 at the time of registration.

CD4 counts at the time of registration

In study group A, only nine subjects had a CD4 count <250 at the time of registration, and 21 subjects had a CD4 count >500, in contrast to group B, where only six subjects had a CD4 count <250 (Table 1).

**Table 1:** Characteristics of study population

Characteristic	Group A	Group B
	n=67 (percentage)	n=66 (percentage)
Age		
21-24 years	22 (32.8)	28 (42.4)
25-29 years	36 (53.7)	25 (37.9)
30-35 years	7 (10.4)	11 (16.7)
35-40 years	2 (2.9)	2 (3.03)
Parity		
Nullipara	31 (46.2)	22 (33.3)
Primipara	23 (34.3)	27 (40.9)
Multipara	12 (17.9)	15 (22.7)
Grand multipara (>4)	1 (1.49)	2 (3.03)
Gestation age at diagnosis		
<12 weeks	2 (2.98)	18 (27.2)
12-23 weeks	20 (29.9)	18 (27.2)
24-35 weeks	25 (37.3)	23 (34.8)
>36 weeks	20 (29.9)	7 (10.4)
Mode of delivery		
Cesarean section	34 (50.7)	35 (66.1)
Vaginal delivery	33 (49.3)	18 (33.9)
Characteristic	Group A	Group B
	n=67 (percentage)	n=66 (percentage)
Birthweight at delivery		
1-1.5 kg	0	1 (1.8)
1.5-2 kg	1 (1.5)	3 (5.7)
2-2.5 kg	25 (37.3)	15 (28.3)
>2.5 kg	41 (61.2)	34 (64.2)
Type of feeding		
Exclusive breast-feeding	55 (82.1)	35 (66.1)
Top feeding	12 (17.9)	18 (33.9)
CD4 count at the start of therapy		
<250	9 (13.4)	6 (11.3)
251-350	13 (19.4)	7 (13.2)
351-500	24 (35.8)	19 (33.8)
>500	21 (31.3)	21 (39.6)

The mean gestational age at which the ART was started in group A was 23±10.73 weeks, while in group B it was 29.3±9.64 weeks. Mean gestational ages at delivery were almost similar in both groups. The average weight of the infant in group A was 2.75 kg, while in group B it was 2.6 kg. The mean CD4 count at the start of ART in group A and group B was 470.76 ±195.92 and 467.87±184.12, respectively.

Table 2 shows the comparison of TLD and TLE group characteristics. 

**Table 2:** Comparison of characteristics of the TLD and the TLE group

	TLD (mean ± SD)	TLE (mean ± SD)
No. Of HIV positive women	67	66
Gestational age at start of therapy (weeks)	23 ± 10.73	29.3 ± 9.64
CD4 count at the start of therapy	470.76 ± 195.92	467.87 ± 184.12
Gestational age at delivery (weeks)	37.13 ± 4.07	37.5 ± 2.38
Birth weight (kg)	2.75 ± 0.39	2.59 ± 0.46

Characteristics of HIV positive infants

None of the infants tested positive during follow-up in group A, while in group B, two infants tested positive, one at six weeks of age and another at 12 months of age. Both of these infants were delivered through cesarean section at 38 and 40 weeks of GA, respectively (Table 3).

**Table 3:** Characteristics of HIV-positive infants

Gestation at the start of TLE (weeks)	CD4 count	Gestation at delivery (weeks)	Mode of delivery	Feeding practices	Positive at age
37	530	38	Cesarean section	Formula feeding	6 weeks
8	371	40	Cesarean section	Breast feeding	12 months

The mother of the first infant, who was tested at six weeks, was detected HIV positive and started taking TLE at 37 weeks of gestation, which is in stark contrast with the other infant, whose mother started taking TLE quite early in her pregnancy, in the first trimester at eight weeks only.

Comparison of neonatal outcome and rate of MTCT in both groups

When results from both groups were compared, it was found that 55 subjects (82%) from group A had healthy neonates, while only 33 HIV-positive mothers (68%) in group B had healthy neonatal outcomes. But this difference was not found to be statistically significant (p-value being 0.13).

None of the infants tested positive for HIV in group A, but in group B, two infants tested positive during follow-up. A vertical transmission rate was observed to be 4.16% in group B, and the difference in vertical transmission rate between the two groups was not found to be statistically significant (χ²= 2.816; p=0.09). This analysis includes only mother-infant pairs with complete 18-month follow-up (therefore, TLD n=67, TLE n=48) (Table 4).

**Table 4:** Effect of EFV- and DTG-based regimens on MTCT of HIV

	TLD (n=67)	TLE (n=48)	p-value
Neonatal outcome (Healthy)	55	33	χ²=4.047 p=0.13
Low birth weight (<2.5kg)	12	15		
HIV positive status	0	2	χ²= 2.816 p=0.09
Vertical transmission rate (%)	0%	4.16%		

CD4 count before and after DTG-based first-line ART in group A

Mean CD4 count at the time of starting TLD was 444.5±199.79, but an overall decreasing trend was observed in CD4 count after six months of TLD therapy, with a mean CD4 count of 405.09±147.52, although the difference has not been found to be statistically significant. And also, comparable follow-up CD4 count data were not available for the TLE group; therefore, intergroup comparison could not be performed.

## Discussion

As we have discussed earlier, in the absence of antiretroviral therapy, the estimated risk of vertical transmission of HIV ranges from 15% to 45%. Administration of a single dose of nevirapine to the mother and nevirapine prophylaxis to the newborn has historically reduced transmission to 10% or less. The introduction of combination ART, along with newborn post-exposure prophylaxis as recommended by WHO in 2013, has the potential to further decrease transmission rates to below 5% among breastfeeding populations [8]. In our previous study, the MTCT rate following the use of the TLE regimen was 4.65% [9], consistent with findings from Dr. S. Sumithra et al., who reported a transmission rate of 6.7% among 74 HIV-exposed infants born to mothers receiving TLE, of whom five infants tested positive [10].

In the present study, the DTG-based regimen (TLD) demonstrated a clear reduction in MTCT, as no infants in the TLD group tested positive. This contrasts with the Botswana study, which reported no significant difference in MTCT between DTG- and EFV-based regimens. A plausible explanation for this discrepancy lies in the timing of infant HIV testing. The Botswana study assessed infants within 96 hours of birth, whereas our study followed infants longitudinally at six weeks, six months, 12 months, and 18 months, allowing late seroconversion, especially via breastfeeding, to be captured [11].

However, the mean gestational age at initiation of ART was earlier in the TLD group, which is a known determinant of MTCT risk and may have influenced the observed outcomes independent of regimen type.

Historically, cesarean delivery was presumed to lower MTCT risk. However, in our study, both infants who acquired HIV in the TLE group were born via cesarean section, indicating that mode of delivery did not significantly influence transmission when maternal viral suppression was inadequate.

Among the two HIV-positive infants in Group B, the first mother was newly diagnosed at 37 weeks and delivered within a week. The short interval between ART initiation and delivery likely did not permit adequate viral suppression. This infant, although formula-fed, tested positive at six weeks, suggesting in utero or intrapartum transmission. The second mother commenced TLE at eight weeks of gestation and delivered at term. Her infant, who was breastfed, tested positive at 12 months despite receiving nevirapine prophylaxis during follow-up.

This observation differs from the findings of Wolde Facha et al., who reported that infants receiving mixed feeding before six months were 1.83 times more likely to acquire HIV compared to those who were exclusively breastfed [12]. Evidence suggests that the gut microbiome of exclusively breastfed infants is dominated by protective bacterial species, promoting mucosal integrity [13]. 

Limitations

However, this study has certain limitations. Being retrospective, it relied on previously recorded data. The comparison between TLE and TLD regimens was conducted across two different time periods, introducing a potential temporal confounding, and improvements in PMTCT services, counselling, and healthcare systems over time may have influenced outcomes independent of the regimen used. As this is a single-center study, results may not reflect outcomes in diverse healthcare settings. 

Future research should include larger multicenter studies to validate these findings. Strengthening PMTCT programs with improved follow-up and counselling can further reduce pediatric HIV transmission.

## Conclusions

Pediatric HIV infections resulting from parent-to-child transmission continue to pose a significant public health challenge in India. Strengthening access to antiretroviral therapy and enhancing the quality of maternal and newborn care are crucial steps toward achieving the goal of virtual elimination of pediatric HIV.

In this study, the DTG-based (TLD) regimen showed a trend toward lower MTCT compared to the EFV-based (TLE) regimen. These findings support the continued scale-up and integration of TLD as the preferred regimen to meet national and global targets for eliminating new HIV infections among exposed infants.
